# Symptomatic and asymptomatic venous thromboembolism after minimally invasive surgery for gynecological cancers

**DOI:** 10.1111/jog.70054

**Published:** 2025-08-23

**Authors:** Michiko Kubo‐Kaneda, Hanano Hirota, Saki Kotaka, Asumi Okumura, Tsuyoshi Mastumoto, Kota Okamoto, Masafumi Nii, Kenta Yoshida, Kuniaki Toriyabe, Eiji Kondo

**Affiliations:** ^1^ Department of Obstetrics and Gynecology Mie University School of Medicine Tsu‐city Japan; ^2^ Faculty of Medicine Mie University Graduate School of Medicine Tsu‐city Japan

**Keywords:** anticoagulant, cancer, gynecology, pulmonary embolism, venous thrombosis

## Abstract

**Aims:**

To clarify the frequency of postoperative symptomatic and asymptomatic venous thromboembolism (VTE) in patients who underwent minimally invasive surgery (MIS) for gynecological cancers; and to identify the risk factors associated with pulmonary embolism (PE).

**Methods:**

We analyzed data for patients with endometrial, cervical, or ovarian cancers who underwent MIS or open surgery between February 2012 and December 2021 at Mie University Hospital, Japan. Patients who required conversion to open surgery were excluded. We treated deep vein thrombosis (DVT), including distal DVT, with preoperative anticoagulation. In all cases, intra‐ and postoperative VTE prophylaxis with anticoagulation, intermittent pneumatic compression, and compression stockings were provided.

**Results:**

Overall, 382 patients with gynecological cancers who underwent MIS were included. Approximately 90% of patients had stage I disease. Symptomatic and asymptomatic PE occurred in 0.2% and 1.5% of patients who underwent MIS, respectively. All patients who developed PE had DVT. In the MIS group, both DVT and PE occurred in seven cases each (1.8%). Conversely, in the open surgery group (*n* = 817), there were 19 (2.3%) and 13 (1.6%) cases of DVT and PE, respectively. DVT and PE incidence rates did not significantly differ between the MIS and open surgery groups (DVT: *p* = 0.67, PE: *p* = 0.80). Uni‐ and multivariate analyses revealed that an operative time >6 h was associated with PE (*p* = 0.034).

**Conclusions:**

VTE incidence was low among patients with gynecological cancers who underwent MIS. VTE rates remained low following open surgery or MIS when appropriate anticoagulation was administered. However, caution should be exercised during prolonged surgeries.

## INTRODUCTION

Venous thromboembolism (VTE), encompassing pulmonary embolism (PE) and deep vein thrombosis (DVT), is a potentially fatal perioperative complication. The incidence of VTE is particularly high among patients with gynecological malignancies, with reported rates of 27%, 11.5%, and 7.3% for ovarian, endometrial, and cervical cancers, respectively.^1^ Advanced age, malignancy, intraoperative lithotomy position, and pelvic surgery are established risk factors for VTE in patients undergoing gynecological surgery.^2^, ^3^, ^4^ The number of minimally invasive surgeries (MISs) performed for endometrial and cervical cancers has rapidly increased. The American College of Obstetricians and Gynecologists recommends 2–4 weeks of perioperative anticoagulation in patients undergoing any procedure for a gynecologic malignancy,^5^ whereas the American College of Chest Physicians suggests a duration of 4 weeks.^6^ However, the duration of anticoagulation is often reduced in cases of MIS due to the possibility of early discharge. Anticoagulant therapy following MIS for gynecological malignancies has not been established.

Recent advances in computed tomography (CT) have enabled the diagnosis of peripheral and asymptomatic PE (APE). Although the prognosis of APE is uncertain, with no consensus on its treatment, APE may exert the same prognostic impact as that of symptomatic PE (SPE).^7^ Reports on the prevalence of APE following MIS, particularly for gynecological cancers, remain scarce. In this study, we aimed to clarify the frequency of symptomatic and asymptomatic VTE following MIS for gynecological cancers and identify the risk factors associated with PE.

## METHODS

### Patients

This retrospective study was approved by the Ethics Committee of Mie University Hospital (approval no. H2023‐191; October 4, 2023) and adhered to the standards of the Declaration of Helsinki, as revised in 2001. Informed consent was obtained using an opt‐out method available on the website. We included patients with endometrial, cervical, and ovarian cancers who underwent MIS between February 2012 and December 2021 at Mie University Hospital. MIS for ovarian cancer was conducted as a part of a clinical trial and self‐funded procedure, following ethics committee approval. As a control group, we included patients with endometrial, cervical, and ovarian cancers who underwent open surgery during the same period. MIS has been implemented since 2013 for stage I endometrial cancer, since 2016 for stage I–IIA cervical cancer, and since 2021 for stage I ovarian cancer. Cases involving uterine morcellation, cervical cancer with tumors ≥2 cm (since 2019), ovarian tumors ≥10 cm, or advanced‐stage disease ineligible for MIS were managed with open surgery under informed consent. The World Health Organization Committee classification of tumors^8^ was used to classify the patients. Patients who required conversion to open surgery were excluded from the study.

### Clinical and pathological data

Clinical data, including age, medical history, stage, surgical procedure, preoperative and postoperative VTE, were obtained from the patients' medical records. Postoperative VTE was diagnosed through contrast‐enhanced CT or ultrasound and was defined as the presence of a new or enlarged pre‐existing thrombus within 1 month following surgery. SPE was defined as the presence of symptoms such as dyspnea, chest pain, syncope, and tachycardia. Symptomatic DVT was identified by the presence of leg swelling and pain. The primary outcome of the study was the incidence of postoperative VTE, encompassing both PE and DVT.

### VTE management

**FIGURE 1:**
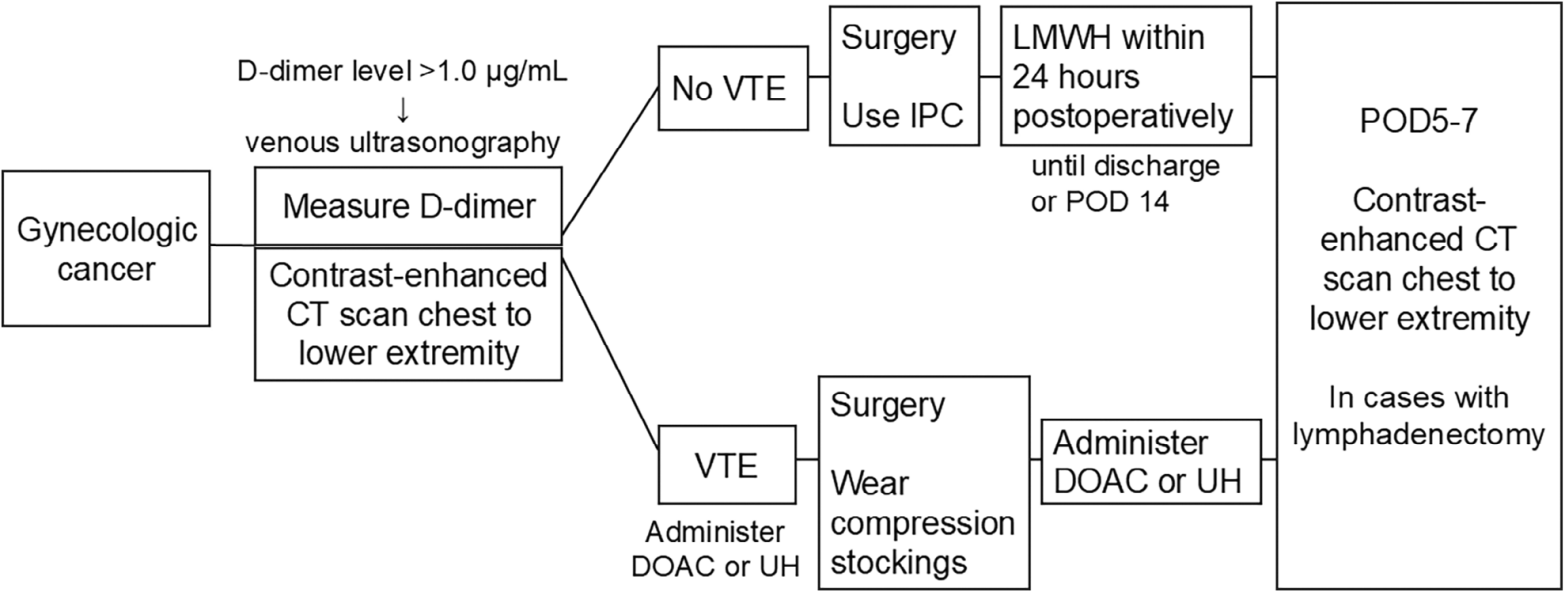
Management of venous thromboembolism (VTE). CT, computed tomography; DOAC, direct oral anticoagulant; UH, unfractionated heparin; IPC, intermittent pneumatic compression; LMWH, low‐molecular‐weight heparin; POD, postoperative day.

Figure 1 shows the perioperative management of patients with cancer who underwent anticoagulation therapy. The D‐dimer levels were measured in all patients at the initial visit; when the D‐dimer level was higher than 1.0 μg/mL, venous ultrasonography was performed to assess the morphology of the thrombus and to provide a baseline for comparison during follow‐up evaluations. Preoperative PE and DVT were periodically assessed in all patients using contrast‐enhanced CT from the chest to lower extremities. If VTE (including PE and distal DVT) was diagnosed postoperatively, treatment was initiated with direct oral anticoagulant (DOAC) or unfractionated heparin (UH) administration. Patients received DOACs as either an initial apixaban (10 mg twice daily) or edoxaban (30 or 60 mg once daily) as the initial dose. Intermittent pneumatic compression was applied intraoperatively and postoperatively in all patients except those with DVT. The patients with DVT wore compression stockings. In patients without VTE, enoxaparin (2000 IU) was administered subcutaneously every 12 h, starting 24 h postoperatively, and continued until discharge or postoperative day (POD) 14. In cases of DVT, UH was resumed 6 h postoperatively, and DOACs were restarted 48 h postoperatively. In all patients who underwent lymphadenectomy, contrast‐enhanced CT from the chest to the lower extremities, including the early arterial phase, was performed on POD 5–7. Patients without lymphadenectomy underwent contrast‐enhanced CT only if symptoms suggestive of thrombosis developed. Anticoagulant therapy for VTE was administered until 3 months after confirming the disappearance of the thrombus using ultrasound.

### Surgical procedure

General and epidural anesthesia was used in all cases, excluding heparin‐administered cases. Surgeries were performed in the lithotomy position using a boot‐type fixator. The angles of pelvic elevation were 15° and 25° for the laparoscopic and robotic surgeries, respectively. The lithotomy and head positions were released approximately every 3.5 h. We performed laparoscopic para‐aortic lymphadenectomy using a combined retroperitoneal and transperitoneal approach, while pelvic lymphadenectomy was conducted via a transperitoneal approach.

### Statistical analysis

Statistical analyses were performed using Fisher's exact and Mann–Whitney U tests. The hazard ratio (HR) and associated 95% confidence interval (CI) were calculated using a stratified Cox proportional hazards model to identify the risk factors associated with PE following MIS. Factors included in the uni‐ and multivariate analyses were age, body mass index, medical history associated with thrombosis (e.g., diabetes mellitus, hypertension, and hyperlipidemia), operative time, blood loss, para‐aortic lymphadenectomy, and preoperative DVT. Statistical significance was set at *p* <0.05. All statistical analyses were conducted using SPSS, version 28.0 (IBM Corp., Armonk, NY, USA).

## RESULTS

We included 310 patients with endometrial cancer, 69 patients with cervical cancer, and 3 patients with ovarian cancer who underwent MIS (median age, 56 vs. 46 vs. 45 years; stage I disease rate, 89% vs. 82.6% vs. 100%). The patients' clinical data are presented in Table 1.

**TABLE 1:** Clinical data of patients included in the study.

	Endometrial cancer (*n* = 310)	Cervical cancer (*n* = 69)	Ovarian cancer (*n* = 3)
Age (years)	56 (29–90)	46 (28–79)	45 (34–52)
Body mass index (kg/m^2^)	24.8 (13.3–67.7)	21.2 (15.1–32.7)	21.1 (18.4–27.6)
Medical history associated with thrombosis^a^	138 (44.4%)	13 (18.8%)	1 (33.3%)
FIGO stage			
I	276 (89.0%)	57 (82.6%)	3 (100%)
II	15 (4.8%)	7 (10.6%)	‐
III	17 (5.5%)	4 (5.8%)	‐
IV	2 (0.6%)	1 (1.4%)	‐
Preoperative DVT	13 (41.9%)	2 (2.8%)	‐

Abbreviations: DVT, deep vein thrombosis; FIGO, International Federation of Gynecology and Obstetrics.

^a^ Diabetes mellitus, hypertension, hyperlipidemia.

The median operative time was 253, 372, and 289 min; lymph node dissection was performed in 52.5%, 81.1%, and 100% of patients with endometrial, cervical, and ovarian cancers, respectively (Table 2).

**TABLE 2:** Treatment measures and surgical findings.

	Endometrial cancer (*n* = 310)	Cervical cancer (*n* = 69)	Ovarian cancer (*n* = 3)
Operating time (min)	253 (106–580)	372 (104–480)	289 (207–452)
Volume of blood loss (mL)	8 (2–1454)	151 (5–1773)	180 (10–300)
Robotic surgery	153 (49.4%)	11 (15.9%)	‐
Laparoscopic surgery	157 (50.6%)	58 (84.1%)	3 (100%)
Surgery			
TH + BSO + PEN+PAN±OME	44 (14.1%)	1 (1.4%)	1 (33.3%)
TH + BSO + PEN±OME	119 (38.4%)	55 (79.7%)	‐
TH + BSO ± OME	120 (38.7%)	6 (8.7%)	‐
PAN	27 (8.7%)	7 (10.1%)	2 (66.7%)
Blood transfusion	7 (2.2%)	3 (4.3%)	‐
Postoperative stay (days)	5 (3–37)	8 (4–22)	6 (5–11)

Abbreviations: BSO, bilateral salpingo‐oophorectomy; OME, omentectomy; PAN, para‐aortic lymphadenectomy; PEN, pelvic lymphadenectomy; TH, total hysterectomy.

Table 3 presents postoperative VTE incidence rates. Postoperative PE occurred in 1.6% and 2.8% of patients with endometrial and cervical cancer, respectively. Furthermore, SPE and APE occurred in one (0.2%) and six patients (1.5%), respectively, among those who underwent MIS. DVT occurred in seven cases (1.8%), and all patients with PE had DVT. Of the 15 patients who had pre‐operative VTE, postoperative PE occurred in one case. SPE was treated with UH, whereas APE was treated with DOACs.

**TABLE 3:** Postoperative VTE incidence.

	Endometrial cancer		Cervical cancer		Ovarian cancer	
	MIS *n* = 310	Open *n* = 304	MIS *n* = 69	Open *n* = 206	MIS *n* = 3	Open *n* = 307
DVT	5 (1.6%)	5 (1.6%)	2 (2.8%)	5 (2.4%)	‐	9 (2.9%)
Symptomatic DVT	‐	2 (0.6%)	‐	1 (0.5%)	‐	2 (0.6%)
Asymptomatic DVT	5 (1.6%)	3 (0.9%)	2 (2.8%)	4 (1.9%)	‐	7 (2.3%)
PE	5 (1.6%)	3 (0.9%)	2 (2.8%)	3 (1.5%)	‐	7 (2.3%)
Symptomatic PE	‐	1 (0.3%)	1 (1.4%)	‐	‐	2 (0.6%)
Asymptomatic PE	5 (1.6%)	2 (0.6%)	1 (1.4%)	3 (1.5%)	‐	5 (1.6%)

Abbreviations: DVT, deep vein thrombosis; MIS, minimally invasive surgery; PE, pulmonary embolism; VTE, venous thromboembolism.

In contrast, the incidence of VTE was examined in 304, 206, and 307 patients with endometrial, cervical, and ovarian cancer, respectively, who underwent open surgery between February 2012 and December 2021. In the endometrial cancer group, five (1.6%), one (0.3%), and two (0.6%) cases of DVT, SPE, and APE, respectively, were observed. In the cervical cancer group, five cases of DVT (2.4%) and five cases of APE (1.6%) were observed. In the ovarian cancer group, 10 (3.2%), two (0.6%), and five (1.6%) cases of DVT, SPE, and APE, respectively, were observed. No significant difference in the incidence of DVT (*p* = 0.67) or PE (*p* = 0.80) was observed between the open surgery and MIS groups. Moreover, no significant difference in the incidence of VTE was observed between the open surgery and MIS groups (Table 3). Uni‐ and multivariate analyses revealed a significant association between the operative time >6 h (HR, 5.234; 95% CI, 1.136–24.120; *p* = 0.034) and PE (Table 4). Only operating time was retained in the final multivariate model after stepwise (Wald) selection; no other variables were included.

**TABLE 4:** Uni‐ and multivariate analyses of PE for patients with MIS.

	Univariate analysis			Multivariate analysis		
	HR	95% CI	*p*‐value	HR	95% CI	*p*‐value
Age >65 years (*n* = 80) versus <65 years (*n* = 302)	1.523	0.290–8.000	0.619	−	−	−
Body mass index >25 kg/m^2^ (*n* = 167) versus <25 kg/m^2^ (*n* = 215)	0.965	0.213–4.371	0.963	−	−	−
Medical history associated with thrombosis^a^ Yes (*n* = 152) versus no (*n* = 230)	0.600	0.115–3.133	0.545	−	−	−
Operating time ≥6 h (*n* = 50) versus <6 h (*n* = 332)	5.234	1.136–24.120	0.034	5.234	1.136–24.120	0.034
Volume of blood loss >100 mL (*n* = 122) versus <100 mL (*n* = 260)	2.904	0.640–13.181	0.167			−
Para‐aortic lymphadenectomy Yes (*n* = 83) versus no (*n* = 299)	2.766	0.607–12.610	0.189	−	−	−
Preoperative DVT Yes (*n* = 15) versus no (*n* = 367)	4.298	0.484–38.143	0.191	−	−	−

Abbreviations: CI, confidence interval; DVT, deep vein thrombosis; HR, hazard ratio; MIS, minimally invasive surgery; PE, pulmonary embolism.

^a^ Diabetes mellitus, hypertension, hyperlipidemia.

## DISCUSSION

VTE occurred in 1.8% of patients who underwent MIS (SPE in 0.2%, APE in 1.5%, and DVT in 1.8%). No significant difference in the incidence of VTE was observed between the open surgery and MIS groups. Multivariate analysis revealed that an operative time of >6 h was associated with PE after MIS.

Cancer is a strong risk factor for thrombosis. The Cancer‐VTE Registry, a prospective, nationwide cohort study conducted in Japan, reported a higher incidence of VTE at every stage in cases of gynecological cancers than in other cancers.^9^ The incidence rate of VTE following MIS for gynecologic malignancies is approximately 0.5%–1%,^10^, ^11^, ^12^ and no significant differences in VTE incidence have been reported among MIS techniques, including robot‐assisted, multiport laparoscopy, and single‐port laparoscopy techniques.^13^

Recent advancements in leg ultrasonography have enabled frequent diagnosis of DVT of the lower extremities, including isolated distal DVT (iDDVT). The prognosis of cancer‐related iDDVT has been reportedly similar to that of cancer‐related isolated proximal DVT and worse than that of iDDVT without cancer.^14^ For cancer‐associated distal DVT, anticoagulation is recommended by National Comprehensive Cancer Network guidelines.^15^

Asymptomatic iDDVT in patients with cancer was treated preoperatively with DOACs or UH. In the present study, the incidence rate of symptomatic VTE was 0.2%, similar to that reported in previous studies.^10^, ^11^, ^12^ Moreover, APE was 7.5 times more likely to occur than SPE.

Submassive PE is also reportedly associated with mortality.^7^, ^16^ Cancer‐associated incidental pulmonary embolism (IPE) exhibits high recurrence and mortality rates,^17^ and anticoagulant therapy has been suggested for cancer‐related IPE.^18^ The safety profile of anticoagulation for asymptomatic VTE has been suggested to be favorable.^19^ However, currently, in Japan, no established treatment strategies exist for asymptomatic, cancer‐related PE. Notably, incidental PE occurred in the MIS group in the present study.

A low incidence of VTE has been reported following MIS for gynecological cancers compared with that following open surgery.^20^, ^21^ Similarly, in most general surgical procedures, the frequency of perioperative VTE is reportedly lower following laparoscopic surgery than that after open surgery.^22^, ^23^ The incidence rate of VTE following open surgery has been reported to be 2.6%–5%.^21^, ^24^ In the present study, the incidence rates of VTE in open surgery and symptomatic VTE were low (2.3% and 0.7%, respectively), and no difference in the incidence rate was observed between open surgery and MIS. We intensified anticoagulation therapy based on our previous review of DVT occurrence after open gynecologic cancer surgery.^25^ Adequate anticoagulation therapy may reduce the incidence of VTE after open surgery. Therefore, reducing the time spent in the lithotomy position intraoperatively is recommended to decrease the incidence of DVT.

In patients with gynecological cancers, advanced tumor stage, obesity, history of VTE, prolonged operative time, increased intraoperative blood loss, and prolonged hospital stay are risk factors for VTE.^26^, ^27^ In the present study, only operative time >6 h was an independent risk factor for VTE, and other risk factors might not have been relevant because of appropriate anticoagulation. The lithotomy position is a risk factor for VTE, and if prolonged surgery is expected, PE may be prevented by releasing the lithotomy position in a short time. Edoxaban, a DOAC used for perioperative thromboprophylaxis, is beneficial in orthopedic surgery,^28^ and this treatment is covered by insurance in Japan. The VALERIA trial,^29^ a randomized controlled trial, reported that rivaroxaban, another DOAC, might be an effective alternative to enoxaparin for perioperative thromboprophylaxis in gynecological cancer surgery. DOACs may be effective for perioperative thromboprophylaxis in gynecological cancer surgeries with a high thrombotic risk. After discharge, DOACs may be necessary for patients undergoing MIS with operating times >6 h. In Japan, prospective trials evaluating DOAC use for thromboprophylaxis in cases of gynecological cancer surgery are needed.

The present study had certain limitations. First, it was a retrospective study. However, appropriate data collection and statistical methods were used to minimize potential bias. Second, APE might not have been detected in patients who did not undergo CT. However, we considered that prolonged operative time was a risk factor for VTE and that patients who did not undergo CT exhibited a low risk of developing VTE because of the short operation time.

In conclusion, the incidence rate of VTE was low in patients with gynecological cancer who underwent MIS. The incidence rate of VTE following open surgery or MIS performed with appropriate anticoagulation therapy was low; however, caution should be exercised during prolonged surgery. The present results may help predict perioperative VTE outcomes of MIS.

## AUTHOR CONTRIBUTIONS

**Michiko Kubo‐Kaneda:** Conceptualization; writing – original draft; data curation; formal analysis; writing – review and editing; investigation. **Hanano Hirota:** Data curation; investigation. **Saki Kotaka:** Investigation. **Asumi Okumura:** Investigation. **Tsuyoshi Mastumoto:** Investigation. **Kota Okamoto:** Investigation. **Masafumi Nii:** Investigation. **Kenta Yoshida:** Investigation. **Kuniaki Toriyabe:** Investigation. **Eiji Kondo:** Conceptualization; investigation; data curation; writing – original draft; writing – review and editing; supervision.

## FUNDING INFORMATION

This research did not receive any specific grant from funding agencies in the public, commercial, or not‐for‐profit sectors.

## CONFLICT OF INTEREST STATEMENT

The authors declare no conflicts of interest.

## Data Availability

We have uploaded the original data in an independent public data repository and it is accessible at https://zenodo.org/uploads/15865667.

## References

[1] Tasaka N , Minaguchi T , Hosokawa Y , Takao W , Itagaki H , Nishida K , et al. Prevalence of venous thromboembolism at pretreatment screening and associated risk factors in 2086 patients with gynecological cancer. J Obstet Gynaecol Res. 2020;46:765–773. 10.1111/jog.14233 32147891

[2] Agnelli G . Prevention of venous thromboembolism in surgical patients. Circulation. 2004;110:IV4–I12. 10.1161/01.CIR.0000150639.98514.6c 15598646

[3] Geerts WH , Bergqvist D , Pineo GF , Heit JA , Samama CM , Lassen MR , et al. Prevention of venous thromboembolism: American College of Chest Physicians Evidence‐Based Clinical Practice Guidelines (8th edition). Chest. 2008;133:381S–453S. 10.1378/chest.08-0656 18574271

[4] Agnelli G , Bolis G , Capussotti L , Scarpa RM , Tonelli F , Bonizzoni E , et al. A clinical outcome‐based prospective study on venous thromboembolism after cancer surgery: the @RISTOS project. Ann Surg. 2006;243:89–95. 10.1097/01.sla.0000193959.44677.48 16371741 PMC1449979

[5] Committee on Practice Bulletins , Gynecology, American College of Obstetricians and Gynecologists . ACOG practice bulletin No. 84: prevention of deep vein thrombosis and pulmonary embolism. Obstet Gynecol. 2007;110:429–440. 10.1097/01.AOG.0000263919.23437.15 17666620

[6] Gould MK , Garcia DA , Wren SM , Karanicolas PJ , Arcelus JI , Heit JA , et al. Prevention of VTE in nonorthopedic surgical patients: antithrombotic therapy and prevention of thrombosis, 9th ed. American College of Chest Physicians evidence based clinical practice guidelines. Chest. 2012;141(2 Suppl):e227S–e277S. 10.1378/chest.11-2297 22315263 PMC3278061

[7] Jaff MR , McMurtry MS , Archer SL , Cushman M , Goldenberg N , Goldhaber SZ , et al. Management of massive and submassive pulmonary embolism, iliofemoral deep vein thrombosis, and chronic thromboembolic pulmonary hypertension: a scientific statement from the American Heart Association. Circulation. 2011;123:1788–1830. 10.1161/CIR.0b013e318214914f 21422387

[8] Kurman RJ , Carcangiu ML , Herrington CS , Young RHE , editors. WHO classification of tumours of female reproductive organs. Lyon: IARC; 2014.

[9] Ohashi Y , Ikeda M , Kunitoh H , Sasako M , Okusaka T , Mukai H , et al. Venous thromboembolism in cancer patients: report of baseline data from the multicentre, prospective cancer‐VTE registry. Jpn J Clin Oncol. 2020;50:1246–1253. 10.1093/jjco/hyaa112 32715307 PMC7579341

[10] Bouchard‐Fortier G , Geerts WH , Covens A , Vicus D , Kupets R , Gien LT . Is venous thromboprophylaxis necessary in patients undergoing minimally invasive surgery for a gynecologic malignancy? Gynecol Oncol. 2014;134:228–232. 10.1016/j.ygyno.2014.05.012 24875122

[11] Nick AM , Schmeler KM , Frumovitz MM , Soliman PT , Spannuth WA , Burzawa JK , et al. Risk of thromboembolic disease in patients undergoing laparoscopic gynecologic surgery. Obstet Gynecol. 2010;116:956–961. 10.1097/AOG.0b013e3181f240f7 20859161

[12] Kumar S , Al‐Wahab Z , Sarangi S , Woelk J , Morris R , Munkarah A , et al. Risk of postoperative venous thromboembolism after minimally invasive surgery for endometrial and cervical cancer is low: a multi‐institutional study. Gynecol Oncol. 2013;130:207–212. 10.1016/j.ygyno.2013.04.024 23612315

[13] Wagar MK , Sobecki JN , Chandereng T , Hartenbach EM , Wallace SK . Postoperative venous thromboembolism in gynecologic oncology patients undergoing minimally invasive surgery: does modality matter? Gynecol Oncol. 2021;162:751–755. 10.1016/j.ygyno.2021.06.011 34148718

[14] Galanaud JP , Sevestre MA , Pernod G , Genty C , Richelet S , Kahn SR , et al. Long‐term outcomes of cancer‐related isolated distal deep vein thrombosis: the OPTIMEV study. J Thromb Haemost. 2017;15:907–916. 10.1111/jth.13664 28266773

[15] National Comprehensive Cancer Network (NCCN) . Clinical Practice Guidelines Oncology Version 2. 2023.

[16] Becattini C , Vedovati MC , Agnelli G . Prognostic value of troponins in acute pulmonary embolism: a meta‐analysis. Circulation. 2007;116:427–433. 10.1161/CIRCULATIONAHA.106.680421 17606843

[17] van der Hulle T , den Exter PL , Planquette B , Meyer G , Soler S , Monreal M , et al. Risk of recurrent venous thromboembolism and major hemorrhage in cancer‐associated incidental pulmonary embolism among treated and untreated patients: a pooled analysis of 926 patients. J Thromb Haemost. 2016;14:105–113. 10.1111/jth.13172 26469193 PMC7480998

[18] Sun JM , Kim TS , Lee J , Park YH , Ahn JS , Kim H , et al. Unsuspected pulmonary emboli in lung cancer patients: the impact on survival and the significance of anticoagulation therapy. Lung Cancer. 2010;69:330–336. 10.1016/j.lungcan.2009.11.015 20007002

[19] Migita S , Okumura Y , Fukuda I , Nakamura M , Yamada N , Takayama M , et al. Rivaroxaban treatment for asymptomatic venous thromboembolism: insights from the J'xactly study. Thromb J. 2023;21:88. 10.1186/s12959-023-00528-w 37599351 PMC10440934

[20] Graul A , Latif N , Zhang X , Dean LT , Morgan M , Giuntoli R , et al. Incidence of venous thromboembolism by type of gynecologic malignancy and surgical modality in the national surgical quality improvement program. Int J Gynecol Cancer. 2017;27:581–587. 10.1097/IGC.0000000000000912 28187092 PMC5539959

[21] Swift BE , Maeda A , Bouchard‐Fortier G . Low incidence of venous thromboembolism after gynecologic oncology surgery: who is at greatest risk? Gynecol Oncol. 2022;164:311–317. 10.1016/j.ygyno.2021.12.011 34920887

[22] Nguyen NT , Hinojosa MW , Fayad C , Varela E , Konyalian V , Stamos MJ , et al. Laparoscopic surgery is associated with a lower incidence of venous thromboembolism compared with open surgery. Ann Surg. 2007;246:1021–1027. 10.1097/SLA.0b013e31815792d8 18043105

[23] Shapiro R , Vogel JD , Kiran RP . Risk of postoperative venous thromboembolism after laparoscopic and open colorectal surgery: an additional benefit of the minimally invasive approach? Dis Colon Rectum. 2011;54:1496–1502. 10.1097/DCR.0b013e31823302a1 22067177

[24] Nguyen JMV , Gien LT , Covens A , van Nguyen JM , Kupets R , Osborne RJ , et al. Dual mechanical and pharmacological thromboprophylaxis decreases risk of pulmonary embolus after laparotomy for gynecologic malignancies. Int J Gynecol Cancer. 2022;32:55–61. 10.1136/ijgc-2020-001205 32571889

[25] Kondo E , Tabata T , Shiozaki T , Motohashi T , Tanida K , Okugawa T , et al. Large or persistent lymphocyst increases the risk of lymphedema, lymphangitis, and deep vein thrombosis after retroperitoneal lymphadenectomy for gynecologic malignancy. Arch Gynecol Obstet. 2013;288:587–593. 10.1007/s00404-013-2769-0 23455541

[26] Barber EL , Clarke‐Pearson DL . Prevention of venous thromboembolism in gynecologic oncology surgery. Gynecol Oncol. 2017;144:420–427. 10.1016/j.ygyno.2016.11.036 27890280 PMC5503672

[27] Sandadi S , Lee S , Walter A , Gardner GJ , Abu‐Rustum NR , Sonoda Y , et al. Incidence of venous thromboembolism after minimally invasive surgery in patients with newly diagnosed endometrial cancer. Obstet Gynecol. 2012;120:1077–1083. 10.1097/aog.0b013e31826c31fb 23090525

[28] AlHajri L , Jabbari S , AlEmad H , AlMahri K , AlMahri M , AlKitbi N . The efficacy and safety of edoxaban for VTE prophylaxis post‐orthopedic surgery: a systematic review. J Cardiovasc Pharmacol Ther. 2017;22:230–238. 10.1177/1074248416675732 27811198

[29] de Longo Oliveira AL , de Oliveira Pereira RF , Agati LB , Ribeiro CM , Kawamura Suguiura GY , Cioni CH , et al. Rivaroxaban versus enoxaparin for thromboprophylaxis after major gynecological cancer surgery: the VALERIA trial: venous thromboembolism prophylaxis after gynecological pelvic cancer surgery with RIvaroxaban versus EnoxAparin (VALERIA trial). Clin Appl Thromb Hemost. 2022;28:10760296221132556. 10.1177/10760296221132556 36474344 PMC9732794

